# Effect of a Diet Supplemented with Malic Acid–Heat (MAH) Treated Sunflower on Carcass Characteristics, Meat Composition and Fatty Acids Profile in Growing Lambs

**DOI:** 10.3390/ani10030487

**Published:** 2020-03-15

**Authors:** Andres Haro, Trinidad de Evan, Jesús De La Fuente Vázquez, María Teresa Díaz, Javier González Cano, María Dolores Carro

**Affiliations:** 1 Departamento de Producción Agraria, ETSIAAB, Universidad Politécnica de Madrid, Ciudad Universitaria, 28040 Madrid, Spain; an.haro@alumnos.upm.es (A.H.); tdeevan@ucm.es (T.d.E.); javier.gonzalez@upm.es (J.G.C.); 2 Departamento de Producción Animal, Facultad de Veterinaria, Universidad Complutense de Madrid, Avda. Puerta de Hierro, s/n. 28040 Madrid, Spain; jefuente@vet.ucm.es; 3 Department of Food Technology, Instituto Nacional de Investigación y Tecnología Agraria y Alimentaria—INIA, Ctra de la Coruña km 7.5, 28040 Madrid, Spain; diaz.teresa@inia.es

**Keywords:** meat quality, lambs, sunflower seeds and meal, fatty acid profile

## Abstract

**Simple Summary:**

One of the main objectives of animal nutrition is to optimize the production of the animals in order to obtain good-quality products, but decreasing the environmental pollution caused by livestock production has also become a priority in the last years. Compared to other farm animals, ruminants have low N utilization efficiency that is mainly due to the inefficient use of nitrogen (N) in the rumen and a large portion of the ingested N is excreted causing environmental problems such as water and soil eutrophication. In this study we analyzed the influence of feeding sunflower seeds (SS) and meal (SM) subjected to a combined malic acid–heat treatment (MAH) on carcass characteristics and meat composition and fatty acid profile of growing lambs. The MAH treatment was applied to reduce ruminal degradation of SS and SM protein and therefore N losses. Compared to control-fed lambs, animals fed the treated SS and SM had increased dorsal fat thickness and yellowness and chromaticity of the Rectus abdominis muscle, which might be beneficial for consumers’ acceptance as they associate a bright meat color with meat freshness and quality. However, there were no differences between groups in meat chemical composition and fatty acid profile.

**Abstract:**

The objective of the study was to assess the effects of feeding sunflower meal (SM) and seeds (SS) protected against rumen degradation on carcass characteristics and composition and fatty acid (FA) profile of lamb meat. The protection of SM and SS was achieved by treating both feeds with malic acid at 150 °C for 2 h (MAH treatment) and in a previous study this treatment was shown to decrease ruminal degradability of protein of both feeds and fat degradability of SS. Two homogeneous groups of 12 lambs each were fed ad libitum high-cereal concentrates and cereal straw from 14 to 26 kg of body weight. The two concentrates differed only in the treatment SM and SS, which were included either untreated (control) or MAH treated. The MAH-fed lambs had greater thickness of dorsal fat (*p* = 0.016) and greater (*p* ≤ 0.016) values of the color parameters a* (redness) and C* (chromaticity) of the Rectus abdominis muscle. However, there were no differences in carcass measurements and in water-holding capacity, chemical composition, pH, color, or fatty acid of Longissimus muscle. In summary, the MAH treatment resulted in only subtle changes in meat composition and quality.

## 1. Introduction

The consumers’ concerns on both the quality of animal products and the polluting emissions of livestock farming have steadily increased in last decades. Compared to other farm animals, ruminants have low N utilization efficiency that is mainly due to the inefficient use of nitrogen (N) in the rumen. As a consequence, a large portion of the dietary N is excreted and contributes to aggravate environmental problems such as water and soil eutrophication. This problem is especially marked when feeds containing high rumen-degradable proteins are fed [1].

Sunflower is one of the most important oilseed crops worldwide and can be used in ruminant feeding either as the whole seed (SS) or as sunflower meal (SM) that is the by-product obtained after the oil extraction. Sunflower protein is rich in sulfur-containing amino acids and thyptophan and low in lysine [2], and therefore its amino acid profile balances well with legume protein, but is about 80% rumen-degradable [1]. Several treatments for protein protection against ruminal degradation have been assessed, but a combined malic acid and heat treatment (MAH) has proven to reduce sunflower protein degradation [3,4]. The in situ degradability of MAH-treated SS and SM was measured in a previous study [5], and the MAH treatment of both feeds increased the rumen-undegradable protein by 19.1% and 120% for SS and SM, respectively, and the rumen-undegradable fat of SS by 34%. As showed by Mejewska et al. [6], feeding lambs with different sources of sunflower fat with varying degree of susceptibility to ruminal biohydrogenation modified the fatty acid (FA) profile of the Longissimus dorsi in a form-dependent manner. We hypothesized that feeding MAH-treated SS and SM to lambs may change the meat FA profile towards a less saturated profile by increasing the duodenal flow of unsaturated sunflower FA. Because both carcass characteristics and meat quality in ruminants are strongly affected by the diet of the animals, the objective of the study was to evaluate the effects of feeding MAH-treated SM and SS on carcass characteristics and meat composition and FA profile of light lambs. This is a companion paper to Haro et al. [7], which reported the effects of the MAH treatment on growth performance, diet digestibility, blood parameters, and ruminal fermentation of growing lambs.

## 2. Materials and Methods

Diets, animals, and experimental procedures were detailed described by Haro et al. [7], and are briefly summarized below. Animals were cared for and managed in accordance with the Spanish guidelines for experimental animal protection, and the experimental procedures were approved by the General Direction of Livestock and Agriculture of the Community of Madrid (Approval number PROEX 035/17).

### 2.1. Diets, Animals, and Experimental Procedure

Two high-cereal concentrates were formulated using the same feed ingredients. The control concentrate contained 264, 263, 196, 109, 89.0, 50.0, 22.4, 4.8, and 2.0 g of barley, corn, wheat, SM, SS, soybean meal, CO_3_Ca, NaCl and mineral-vitamin premix per kg (fresh matter basis), respectively. In the MAH concentrate, both SM and SS were included after being treated for protecting sunflower protein against ruminal degradation. The MAH treatment has been detailed by Haro et al. [7] and consisted in spraying both feeds with a 1 M malic acid solution at a rate of 400 mL per kg of feed and drying the mixture at 150 °C for 2 h. Chemical composition was similar in both concentrates, which contained as average 941, 155, 188, and 69.9 g of organic matter, crude protein, neutral detergent fiber and acid detergent fiber per kg, respectively [6].

Two groups of 12 Lacaune lambs each, weighting as average 14.2 ± 0.35 kg, were individually housed in 1 m × 1 m pens, and fed the experimental concentrates and barley straw ad libitum until reaching 26 kg of body weight (39 days).

### 2.2. Slaughter Measurements and Sampling

Lambs were slaughtered at a commercial abattoir located 30 km away from the experimental farm on two different days (6 lambs of each treatment per day). Feed and water were available until transport the lambs to the abattoir, where they were slaughtered within 2 h by exsanguination after mechanical stunning. The carcasses were weighed immediately after slaughter and after 24 h at 4 °C. Carcass conformation measurements were made as proposed by Cañeque et al. [8]. Briefly, carcass width (CWD) was the widest carcass measurement at the ribs, thoracic depth (CTD) was measured as the maximum distance between the sternum and the back of the carcass at the sixth thoracic vertebra, buttock width (BUW) was the widest buttock measurement in a horizontal plane on the hanging carcass, hind limb length (HLL) was the length from perineum to distal edge of the tarsus, and internal carcass length (ICL) was measured as the length from cranial edge of the symphysis pelvis to the cranial edge of the first rib. Carcass compactness was calculated as (cold carcass weight/ICL) and buttock/leg index was estimated as (BUW/HLL) as proposed by [9]. Carcass fatness was subjectively evaluated using the 1–4 points scoring system following the community scale for classification of lamb carcass [10]. The dorsal fat thickness was measured at 4 cm from the carcass midline and at 4 cm from the caudal edge of the last rib with a digital calibrator [11]. The kidney knob and channel fat (KKCF) score was assessed using a 1 to 3 scale [12]. Carcasses were split down by the dorsal midline, and the left hind leg was separated, dissected and the length and weight of the metacarpus were recorded.

In addition, the following measurements were taken. The pH of the Longissimus and Semitendinosus muscles were measured at 0 and 24 h after slaughter using a penetration electrode adapted to a portable pH meter (Hanna Instruments pH meter HI-9025; Hanna Instruments SL, Eibar, Spain). At each time, two measurements were made for each muscle. Color was measured 24 h after slaughter on the subcutaneous fat of the tail root, on Rectus abdominis muscle and on Longissimus muscle (on the cut surface 1 h after blooming) using a Minolta Spectophotometer CM-2500c (Minolta, Osaka, Japan) with illuminant D65, visual angle of 10° and measurement aperture of 8 mm. Three measurements were made on each location and values were averaged before statistical analysis. The calibration was performed as described by [11] using standard white tiles prior to color measurements. The color coordinates were expressed following the CIELAB system [13] as L* (brightness), a* (red-green index) and b* (yellow-blue index). Chroma (C*) and hue (h*) values were calculated as C* = (a*2 + b*2) 0.5 and h* = tan – 1 (b* / a*), respectively.

The water-holding capacity of meat (Longissimus muscle) was measured as pressure loss using the procedure of [14], and it was expressed as the percentage of expelled juice after compression. A sample of the Longissimus of each lamb (L1–L4) was taken and external fat and connective tissue were removed before being frozen and freeze-dried for analysis of chemical composition and FA profile of meat.

### 2.3. Chemical Analyses

All chemical analyses were performed in duplicate unless otherwise stated. Chemical composition of feeds was analyzed according the Association of Official Analytical Chemists [15] procedures as described by [7].

For moisture analysis of meat, 5 g of the Longissimus muscle were homogenized in a crucible with sea sand, 5 mL of ethanol were added, and the sample was dried at 102 °C. The ash and ether extract content of the meat was analyzed following the Association of Official Analytical Chemists [15] procedures (ID 048.13 and 945.16, respectively), whereas the N content was analyzed by the Dumas combustion method employing a Leco FP258 N Analyzer (Leco Corporation, St. Joseph, MI, USA).

Fatty acid methyl esters (FAME) of freeze-dried Longissimus muscle were prepared (in duplicate) by direct bimethylation according to the method described by [16], and were analyzed using a gas chromatograph (Perkin-Elmer Autosystem-1:A, Waltham, MA, USA) equipped with a flame ionisation detector and an Omegawax 320 capillary column (30 m × 0.32 mm internal diameter; 0.25 mm film thickness) with polyethylene glycol as the stationary phase (Supelco, Bellefonte, PA, USA). Chromatographic conditions had been detailed described by [17]. Individual FAME were identified by comparing their retention times with those from a known standard (Supelco, Bellefonte, PA, USA), and results were expressed as percentage of total FA identified.

### 2.4. Statistical Analyses

Normal distribution of data was assessed by the Shapiro–Wilk test [18]. The data were analyzed as a 1-way ANOVA using the GLM procedure of [19], in which the diet was the main effect and lamb the experimental unit. Significance was declared at *p* < 0.05 and trends at *p* < 0.10.

## 3. Results and Discussion

As reported by [7], the MAH treatment did not affect feed intake, average daily gain and hot and cold carcass weights, which reached 12.7 and 12.4 kg for control and 13.7 and 12.8 kg for MAH-fed group, respectively. There were no effects (*p* ≥ 0.134) of MAH concentrate on cooling losses, carcass conformation measurements and metacarpus weight and length (Table 1), which is in agreement with the lack of differences between groups observed in the carcass weights. The measurements of carcass conformation and metacarpus were similar to those reported in other studies with lambs slaughtered at similar body weight [20,21,22]. The lack of differences in carcass conformation measurements is consistent with the similar composition of the diet and feed intake in both groups of lambs (reported by [7]), as the energy and protein intake are main factors affecting lambs’ growth [22,23].

In a trial conducted with growing lambs, Díaz-Royón et al. [24] applied the MAH treatment to SM and spring peas and observed no effects on the amount of kidney-pelvic and dorsal fat. However, in our study the MAH-fed lambs had greater (*p* = 0.016) dorsal fat thickness compared with the control lambs, which might indicate greater energy retention. In the present study and in that of [24] the same MAH treatment was used, similar diets (high-cereal concentrates and cereal straw) were fed, and lambs had similar initial and final weights. Therefore, the different response observed in the two studies in the dorsal fat might be related to the greater precocity of the Lacaune lambs [8,25] used in our study compared with the lower precocity of the “Entrefino” lambs used by [24].

Table 2 shows the values of muscle pH and color. The pH values of the Longissimus and Semitendinosus muscles measured at 0 and 24 h were within the normal ranges for lamb meat [26], and were similar to those reported for light lambs of different breeds in other studies [8,27]. The color parameters of Longissimus and the subcutaneous fat of the tail root were similar (*p* ≥ 0.107) for both groups, but MAH-fed lambs had greater redness (a*; *p* = 0.010) and chromaticity (C*; *p* = 0.016), and tended to have greater yellowness (b*; *p* = 0.061) of the Rectus abdominis than the control lambs. Meat color is affected by numerous factors such as breed, feeding, age and slaughter weight of the animal, exercise, slaughter stress, and meat storage conditions, among others [20,28]. The color of lamb meat is crucial to ensure customer appeal and contributes strongly to the value of the product [29], as consumers associate a bright meat color with freshness and quality [30]. Although no panel test for evaluating meat color was performed in our study, the greater yellowness (b*) and chromaticity (C*) of the MAH-treated meat might be favorable for consumers acceptance, as when the oxidation of the unsaturated fats takes place the yellow changes to a brown color and consumers associate that with the lack of freshness.

Neither the water-holding capacity of the meat nor its chemical composition were affected (*p* ≥ 0.327) by MAH treatment (Table 3). Chemical composition values were in the range of those reported previously for light lambs of different breeds [31,32,33].

Fatty acid profile of the Longissimus muscle is shown in Table 4. There were no differences (*p* ≥ 0.117) between groups, with the exception of a trend (*p* = 0.055) to a greater proportion of C14:0 in the MAH-fed lambs compared with the control ones. The diet is one of the main factors affecting the meat FA profile [34,35], which has important implications for human nutrition [36]. The meat FA profile in our study was similar to that previously reported for lambs fed diets containing SS [32,37], being the C16:0 and C18:0 the most abundant saturated FA.

Sunflower feeds (SS and SM) contain high amounts of unsaturated FA, especially of C18:1 and C18:2 [32,37] and the inclusion of SS in the diet has been reported to increase the proportion of C18:1, C18:2, polyunsaturated FA (PUFA) and n-6 FA in the meat of lambs [32,37]. In our study, both groups were fed SS, but according to a previous study [5] conducted in sheep fed the same concentrates the MAH-treatment increased the supply of by-pass fat by 34% for SS and therefore a different FA profile of meat might be expected.

The content in monounsaturated FA in the Longissimus in our study (45.3 and 44.2 g/100 g total FA for control and MAH concentrate, respectively) were similar to those reported by [32] for lambs fed SS (43.0 g/100 g total FA). In contrast, PUFA concentrations in our study (19.4 g/100 g total FA for both groups) were greater than those observed by (32; 7.86 g/100 g total FA), despite that the level of SS inclusion in the diet was similar in both studies (80.1 g/kg in the study of [32] and 89.0 g/kg in ours). The greater PUFA proportion in our study might indicate a lower biohydrogenation of PUFA in both groups of lambs, possibly due to the low ruminal pH in all animals (5.17 and 5.26 for control and MAH groups, respectively; *p* = 0.510; reported by [7]. In fact, it has been shown that low ruminal pH values decrease the population of ruminal microorganisms responsible for the isomerization and biohydrogenation of PUFA [38]. Although ruminal pH was not measured in the study of [32], the neutral detergent fiber content of the diets fed to lambs (303 g/kg) was greater than that in our study (188 g/kg), and therefore greater ruminal pH values may be expected. A low biohydrogenation rate in the control group in our study might also help to explain the lack of differences between groups in FA profile, despite of the 34% of increase in the fat by-pass fraction of the MAH-treated SS previously measured by [5]. It has to be taken into account that this study was conducted with sheep fed oat hay and concentrate ant the average ruminal pH in the sheep (5.80 and 5.84 for control and MAH groups; average of 4 measurements taken after feeding) was greater than that in the lambs of the present study and therefore greater ruminal biohydrogenation in the untreated SS may be expected. The high content in C18:2 observed in our study (14.3%) compared with others in which lambs were fed diets including SS (about 5–6 g/100 g total FA; [32,37]) seems to support the hypothesis of low biohydrogenation caused by low pH. As a consequence, the proportion of n-6 FA in our study was greater than that reported in other studies feeding lambs sunflower fat [6,32,37] and the n-6/n-3 ratio reached values above 30. Decreasing the n-6/n-3 ratio in the diet has been considered favorable for human health, although some limitations when considering this ratio have also been risen more recently [39]. However, it should be taken into account that the column used to analyze the meat FA profile was not appropriate to identify biohydrogenation intermediates, as longer columns are necessary to analyze these intermediates [40], and therefore the results on meat FA profile are limited to the FA detected.

## 4. Conclusions

The inclusion of SS and SM treated with malic acid and heat for protecting the protein against ruminal degradation in the concentrate of fattening lambs had no effect on carcass characteristics and meat quality, excepting that increased the amount of dorsal fat and the redness (a*) and chromaticity (C*) of the Rectus abdominis. More studies are needed to investigate the feeding conditions under which this treatment might be useful to improve lamb meat quality.

## Figures and Tables

**Table 1:** Cooling losses, carcass conformation, and fatness measurements, and weight and length of the metacarpus of growing lambs fed barley straw and concentrate containing sunflower meal and seed either untreated (control) or treated with malic acid and heat (MAH).

Item	Concentrate		SEM ^1^	*p =*
	Control	MAH		
Cooling losses (%)	2.63	2.43	0.172	0.590
Carcass conformation (cm) ^2^				
CWD	19.3	19.3	0.34	0.973
CTD	20.9	22.0	0.48	0.134
BUW	53.8	54.1	0.54	0.634
HLL	22.3	22.1	0.35	0.693
RP	16.2	16.1	0.31	0.764
ICL	54.3	55.2	0.61	0.319
Carcass compactness	0.23	0.23	0.003	0.508
Buttock/leg index	2.42	2.46	0.056	0.550
Dorsal fat thickness (cm)	1.82	2.43	0.164	0.016
Carcass fatness ^3^	2.22	2.25	0.160	0.906
Pelvic-kidney fat ^3^	1.83	2.12	0.159	0.200
Metacarpus weight (g)	42.4	43.6	2.390	0.791
Metacarpus length (cm)	12.0	12.2	0.139	0.407

^1^ SEM: standard error of the mean ^2^ CWD: carcass width; CTD: thoracic depth; BUW: buttock width; HLL: hind limb length; RP: rump perimeter; ICL: internal carcass length; carcass compactness was calculated as (cold carcass weight/ICL) and buttock/leg index was estimated as (BUW/HLL). ^3^ Measured according a 1 to 4 points scale (1: minimum score; 4: maximum score).

**Table 2:** pH and color values of different tissues of growing lambs fed barley straw and concentrate containing sunflower meal and seed either untreated (control) or treated with malic acid and heat (MAH).

Item	Concentrate			
	Control	MAH	SEM ^1^	*p* =
pH				
Longissimus				
0 h	6.76	6.77	0.043	0.945
24 h	5.68	5.64	0.033	0.365
Semitendinosus				
0 h	6.59	6.44	0.067	0.130
24 h	5.77	5.75	0.044	0.743
Color				
Subcutaneous fat (tail root)				
Lightness (L*)	64.4	63.0	1.66	0.554
Redness (a*)	1.80	3.36	0.656	0.107
Yellowness (b*)	11.9	12.0	0.99	0.933
Chromaticity (C*)	12.0	12.8	0.97	0.590
Hue* (H*)	67.5	60.1	13.6	0.703
Rectus abdominis				
Lightness (L*)	44.1	42.7	1.27	0.448
Redness (a*)	7.39	10.4	0.739	0.010
Yellowness (b*)	5.66	8.26	0.930	0.061
Chromaticity (C*)	9.62	13.4	1.01	0.016
Hue* (H*)	35.5	37.3	3.65	0.724
Longissimus				
Lightness (L*)	38.5	36.6	1.48	0.385
Redness (a*)	8.60	10.2	0.77	0.147
Yellowness (b*)	12.4	13.3	0.43	0.144
Chromaticity (C*)	15.2	16.9	0.75	0.123
Hue* (H*)	55.8	53.1	1.65	0.264

^1^ SEM: standard error of the mean.

**Table 3:** Water holding capacity and chemical composition of the Longissimus muscle of growing lambs fed barley straw and concentrate containing sunflower meal and seed either untreated (control) or treated with malic acid and heat (MAH).

Item	Concentrate			
	Control	MAH	SEM ^1^	*p =*
Water holding capacity (%)	66.8	67.8	1.00	0.506
Chemical composition (%)				
Moisture	75.5	74.9	0.49	0.474
Protein	20.1	20.6	0.30	0.327
Fat	2.80	3.09	0.508	0.690
Ash	1.06	1.09	0.026	0.516

^1^ SEM: standard error of the mean.

**Table 4:** Fatty acid (FA) profile of the Longissimus muscle of growing lambs fed barley straw and concentrate containing sunflower meal and seed either untreated (control) or treated with malic acid and heat (MAH).

Item	Concentrate			
	Control	MAH	SEM ^1^	*p* =
Fatty acid (% of total fatty acids)				
C10:0	0.10	0.10	0.009	0.781
C12:0	0.26	0.35	0.037	0.117
C14:0	3.23	3.60	0.129	0.055
C15:0	0.51	0.51	0.021	0.940
C16:0	21.5	21.2	0.34	0.553
C17:0	1.50	1.43	0.056	0.380
C18:0	8.18	9.09	0.460	0.177
C20:0	0.07	0.08	0.004	0.455
Total saturated FA	35.3	36.3	0.52	0.181
C14:1	0.15	0.15	0.010	0.763
C16:1	2.02	1.96	0.081	0.623
C17:1	0.92	0.87	0.044	0.430
C18:1	42.2	41.2	0.84	0.431
Total monounsaturated FA ^2^	45.3	44.2	0.89	0.410
C18:2 n-6	14.3	14.3	0.68	0.940
C18:3 n-3	0.15	0.16	0.008	0.808
C20:3 n-6	0.17	0.19	0.013	0.211
C20:4 n-6	4.35	4.29	0.245	0.780
C20:5 n-3	0.06	0.06	0.007	0.780
C22:5 n-3	0.29	0.31	0.023	0.570
C22:6 n-3	0.11	0.13	0.020	0.406
Total polyunsaturated FA	19.4	19.4	0.86	0.970
n-6	18.8	18.8	0.85	0.998
n-3	0.61	0.66	0.044	0.442
n-6/n-3	31.4	29.9	1.88	0.563

^1^ SEM: standard error of the mean; ^2^ all isomers of each monounsaturated FA are included.
